# Tufting Enteropathy: A Review of Clinical and Histological Presentation, Etiology, Management, and Outcome

**DOI:** 10.1155/2020/5608069

**Published:** 2020-09-23

**Authors:** Changzhou Cai, Yishu Chen, Xueyang Chen, Feng Ji

**Affiliations:** Department of Gastroenterology, The First Affiliated Hospital of Zhejiang University School of Medicine, Hangzhou 310003, China

## Abstract

Congenital tufting enteropathy (CTE), also named intestinal epithelial dysplasia, is a rare, autosomal recessive enteropathy with persistent and life-threatening intractable diarrhea early in life. Intractable diarrhea is present independent of breast or formula feeding. Most CTE patients require total parenteral nutrition (TPN), and in severe cases, small bowel transplantation is needed. In the last decade, we have seen remarkable progress in certain aspects, such as the pathogenesis and diagnostic methods of the disease. Rapidly developing molecular analysis techniques have improved the diagnostic methods for CTE and reduced invasive and expensive procedures. Mutations in the gene encoding human epithelial cell adhesion molecule (EpCAM) were identified in the typical form of CTE, which usually exhibits isolated refractory diarrhea. Moreover, the syndromic form of CTE features anal and choanal atresias as well as ophthalmologic signs, which are associated with mutations in the gene encoding Serine Peptidase Inhibitor Kunitz Type 2 (SPINT2). This article reviews CTE disease based on its clinical and histological presentation, etiology and pathogenesis, and management and outcome.

## 1. Introduction

Congenital diarrheal disorders (CDDs) are rare hereditary intestinal diseases that are clinically characterized by the emergence of persistent and life-threatening intractable diarrhea early in life, and most cases are caused by genetic defects [1]. The primary clinical manifestation of CDD is severe chronic diarrhea, which is, in some cases, only part of a series of more complex systemic symptoms [2]. The etiology of CDD mainly includes the following four categories: intestinal epithelial dysfunction, defects of enteroendocrine cells, abnormalities in enterocytes, and anomalies in the regulation of the intestinal immune response [3]. Mechanistically, CDD can be divided into osmotic diarrhea and secretory diarrhea. The former is caused by unabsorbed nutrients in the intestinal lumen that fail to function well in driving fluids into the intestine through osmotic forces. The latter manifests active secretion of electrolytes and water into the intestinal lumen, even during fasting, which is due to abnormalities in NaCl absorption or Cl secretion [4].

Congenital tufting enteropathy (CTE), also named intestinal epithelial dysplasia, is a rare, autosomal recessive enteropathy belonging to secretory CDD [5]. In addition to chronic watery diarrhea that occurs within months after birth, some pediatric CTE patients present symptoms such as choanal atresia and dysmorphic facial features, which are a syndromic form of CTE [2, 6]. Most patients with CTE rely on total parenteral nutrition (TPN) for energy support, while in severe cases, small bowel transplantation is required [7, 8]. CTE is pathologically characterized by *villous* atrophy of the intestinal epithelium, disorganization of surface enterocytes, and crypt hyperplasia [9]. Focal epithelial tufts are typically found in the small intestine and occasionally in the colonic mucosa [10]. These tufts are composed of intestinal epithelial cells with rounding of the plasma membrane, forming a teardrop-like structure [11].

The incidence of CTE is estimated to be 1 in 50,000 to 100,000 live births in Western Europe [11], and the incidence is higher in Middle Eastern families [12]. CTE was first identified in 1994 and is suspected to be an autosomal recessive disease [13]. In 2008, genetic studies on 11 CTE patients identified disease-related mutations in the gene encoding human epithelial cell adhesion molecule (EpCAM), which is located on chromosome 2p21 (47,369,147 to 47,387,027) [7]. The EpCAM gene mutations were later confirmed by subsequent studies on CTE patients [14–16]. Furthermore, mutations in the gene encoding Serine Peptidase Inhibitor Kunitz Type 2 (SPINT2) were associated with a syndromic form of CTE [16, 17].

The low incidence of CTE and the paucity of related studies have caused the disease to remain elusive, and CTE patients still suffer from poor prognosis and low quality of life. As there has been no systematic review of CTE in the past ten years, this article is aimed at providing a global view of the disease, including its clinical and histological presentation, etiology and pathogenesis, and management and outcome.

## 2. Clinical and Histological Presentation

### 2.1. Clinical Presentation

CTE is a rare disorder with approximately 150 patients reported in the literature [12, 13, 16] and is clinically very heterogeneous (Table 1). In terms of family history, parents and ancestors of the patients are unaffected with no similar cases [18], but the patients' siblings or cousins could be affected [7, 16, 19] or healthy [16]. Generally, pregnancy and delivery are uneventful, and polyhydramnios associated with congenital sodium diarrhea (CSD) exhibiting congenital sodium-losing diarrhea caused by deficient sodium-hydrogen exchange [20] is not found. Intractable diarrhea is present in the first few weeks or months after birth, independent of breast or formula feeding. Moreover, diarrhea is watery, secretary, and abundant (>50 mL/kg/d) whether the infant is fed or fasted [21], while in some cases, stool output could be ameliorated by fasting [22]. Due to persistent diarrhea and dysfunction of intestinal absorption, infants usually become irritable and develop moderate to severe dehydration and weight loss [23], eventually leading to impaired growth and [9] failure to thrive [24]. Changes in the formula of full-calorie enteral nutrition, such as carbohydrate-free formula or amino acid-based formula, usually fail to reverse the weight loss caused by diarrhea [23]. In addition to intractable diarrhea, CTE patients could present other gastrointestinal symptoms, such as abdominal distension [9] and vomiting [4].

Newborn screening; blood tests for liver, kidney, and pancreas function; imaging methods; and immunologic, allergic, and extensive laboratory work up could not identify the etiology for CTE patients [25]. Vitamin A levels could be average, while vitamin K levels could be elevated [26]. Testing for infectious diseases, including stool cultures for *Clostridium difficile* toxin, stool ova, rotavirus antigen and parasites, and urine and blood cultures for bacteria and viruses, could not provide a reasonable explanation for the infants' condition [26]. Fecal tests could indicate elevated osmolality, osmotic gap, and quantitative fecal fat [22, 23, 26]. No significant increase was observed in the fecal loss of Na^+^, making CTE different from CSD [27].

CTE patients could also show extraintestinal symptoms, which are defined as a syndromic form of CTE (SCTE) [17]. Among all symptoms, ophthalmologic signs are the most reported, including photophobia [6], cataracts [6], corneal erosions [2], and superficial punctuated keratitis (SPK) [6, 16, 28, 29]. Atresia, such as choanal atresia [2, 16, 28, 29], anal atresia [16, 30], and other atresias [16], is another commonly seen parenteral symptom. Other than ophthalmologic signs and atresia, patients with CTE could have cleft lip and palate [2, 31], dermatological anomalies [16], bone malformations [16], optic nerve coloboma [31], cholestatic liver disease [17], chronic arthritis [12, 32], and skeletal dysplasia [18].

### 2.2. Histological Presentation

In general, infants suspected to have CTE undergo esophagogastroduodenoscopy and colonoscopy examination, during which mucosal biopsies are performed at multiple sites. Histological abnormalities in the intestines of patients with CTE include villous atrophy, basement membrane abnormalities, and disorganization of enterocytes with focal crowding at the villus tips [33].

Villous atrophy is present in all patients but varies in severity. In most patients, typical abnormalities are localized mainly in the surface epithelium, forming focal epithelial “tufts” [13, 18]. These tufts consist of tightly packed enterocytes with rounding of the plasma membrane, shaping a tear-like structure by the cells. Moreover, the adjacent epithelium near the tufts is usually free from apparent abnormalities [13]. Sometimes, such characteristic tufts could be absent in the biopsy of early CTE patients with typical clinical symptoms, while the absence of EpCAM in the epithelium could be confirmed by further immunohistochemical staining for MOC-31 [23, 26]. Additionally, focal enterocyte crowding could also be found in the crypt epithelium, and crypts are often characterized by expansion into pseudocysts and abnormal regeneration with branching. A study on biopsy specimens revealed that when compared with controls, the deposits of heparan sulfate proteoglycan in the basement membrane were abnormal in CTE patients [10]. Relative to the controls, the deposition of laminin at the epithelial lamina propria interface was faint and irregular, whereas the removal of heparan sulfate proteoglycan was large and lamellar, indicating that the epithelial abnormalities originated from the irregularities of the basement membrane. In addition, almost no inflammatory cell infiltration was found in the lamina propria, while in some cases reported elsewhere, the numbers of inflammatory cells could increase in the lamina propria, suggesting that the increase in inflammatory cells is not a criterion for CTE exclusion [34].

## 3. Etiology and Pathogenesis

### 3.1. Mutation of EpCAM in CTE

EpCAM, formerly known as TACSTD1 or TROP1, is a type I transmembrane superficial glycoprotein antigen that is expressed on the basolateral membrane of multiple epithelial cells and plasma cells. This molecule was first discovered in 1979 and was identified as a tumor-specific antigen in several rapidly growing epithelial tumors due to its high expression level [35, 36]. EpCAM has long been described as a molecule involved in the interactions between cells, similar to most other cell adhesion molecules. L929 fibroblasts, a cell line generally incapable of cellular adhesion, form multicellular aggregates when expressing EpCAM, indicating the involvement of EpCAM in homotypic cell-cell interactions [36]. However, recent experiments failed to prove the oligomerization of EpCAM protein in vitro [37] and the role of EpCAM in adhesion in carcinoma cells [38]. Based on these recent observations, the hypotheses that EpCAM requires ligands for adhesion and EpCAM is not a cell adhesion molecule have been proposed. Despite the controversy, studies revealed that EpCAM colocalizes with E-cadherin and directly associates with the tight junction protein claudin-7 [39] and facilitates the formation of the intestinal barrier by recruiting claudins to intercellular junctions [40]. In 2008, by using single nucleotide polymorphism (SNP) genotyping, Sivagnanam et al. first revealed the homozygous G>A substitution at the donor splice site of exon 4 in EpCAM in a family with two children diagnosed with CTE. Decreased expression of EpCAM was demonstrated by immunohistochemistry and Western blot in the intestinal tissues of CTE patients, which revealed the loss-of-function of EpCAM caused by mutations [7].

After a comprehensive analysis of previous reports on CTE and EpCAM, we found 103 mutations in EpCAM from 14 studies (Tables 1 and 2) [7, 12, 14–16, 19, 23, 25, 26, 30, 41–44], including 3 chromosomal deletions, 7 noncoding/splicing mutations, 14 frameshift/truncation mutations, and 11 in-frame deletions or missense mutations. Only four of the mutations were reported in five or more patients: c.498insC (21 patients), c.556-14A>G (15 patients), c.491+1G>A (9 patients), and c.492-2A>G (6 patients). The frameshift mutation (c.498insC) was the most common form of mutation in all CTE patients, bringing in 21 different amino acids followed by premature truncation of the protein (Q167Pfsx21) [12]. Among splicing mutations, c.556-14A>G, an intronic mutation, was the most common, forming a new acceptor splice site within intron 5, thus impairing the splicing of the EpCAM transcript [16]. Recent studies revealed that splicing mutations could result in the absence of EpCAM in the transmembrane domain [45]. In general, most reported mutations (chromosomal deletion, splicing mutations, and truncating mutations) are believed to result in the absence of the EpCAM protein. Accordingly, the protein could not be detected by immunohistochemistry in intestinal biopsies [16, 19, 45]. Moreover, missense mutations were reported to manifest as weak EpCAM immunostaining [16] or the disappearance of EpCAM immunohistochemical signals [9]. Although the protein EpCAM seemed detectable by immunohistochemistry in some cases with missense mutations, it is likely that an abnormal extracellular domain of EpCAM was formed, which might impair the related signaling pathways [16]. Clinically, patients with EpCAM mutations mainly present isolated congenital diarrhea without parenteral symptoms [16].

Initially, the in vivo role of EpCAM was studied in two zebrafish knockout models by retroviral insertion or antisense oligonucleotides, revealing that EpCAM is essential for proper epithelial morphogenesis integrity, otolith formation, skin development [46], and proneuromast deposition [47]. Subsequently, several EpCAM knockout mice were generated to study the function of EpCAM. One study showed that EpCAM plays a critical role in forming functional tight junctions in the intestinal epithelium by recruiting claudin protein [40]. Another EpCAM knockout mouse model constructed by a rigorous gene-trapping approach showed intestinal tufts, villous atrophy, and severe hemorrhagic diarrhea. E-cadherin and *β*-catenin showed a disorganized transition from crypts to villi, leading to the loss of the cell membrane but increasing intracellular accumulation [48]. In addition, conditional knockout mice with EpCAM-deficient epithelial Langerhans cells (LCs) manifested inhibition of migration of EpCAM-deficient LCs from the skin to lymph nodes as well as enhanced contact hypersensitivity responses, suggesting the role of EpCAM in promoting LC migration and modulating intercellular adhesion and cell movement [49]. A novel CTE mouse model based on EpCAM mutations (c.491+1G>A) was developed by Cre-LoxP recombination technology. The study showed decreased expression of both EpCAM and claudin-7 as well as disappearance of their colocalization in EpCAM mutation mice. Accordingly, the EpCAM/claudin-7 complex was destroyed, thus enhancing intestinal permeability and intestinal cell migration [42]. Recently, Wu et al. showed that mutations in SPINT2 could weaken its inhibitory effect on matriptase, leading to efficient cleavage of EpCAM and eventually destabilizing claudin-7 in intestinal epithelial cells. These results demonstrated the role of the SPINT2/matriptase/EpCAM/claudin-7 pathway in the pathogenesis of CTE [50].

### 3.2. Mutation of SPINT2 in CTE

Approximately one-fifth of the reported CTE patients have a “syndromic form” of CTE (Table 1). Characteristically, these patients could show unilateral or bilateral choanal atresia at birth [2], accompanied by ophthalmologic diseases throughout life [6]. Less frequently, superficial punctuated keratitis, anal atresia, cleft lip and palate, dermatological anomalies, and bone malformations could be observed. This form of CTE and the syndromic form of congenital sodium diarrhea (SCSD) were thought to be the same disease because of their clinical similarities and the same mutations of the suspected pathogenic gene, SPINT2 [2, 16, 17]. SPINT2, also called hepatocyte growth factor activator inhibitor type 2 (HIA-2), is a transmembrane protein thought to be involved in epithelial regeneration, as well as in the signaling pathways of NF-*κ*B and TGF-*β* [51]. The genome-wide linkage scan first identified a homozygous splice site mutation of SPINT2 in 16 syndromic CTE patients in 2009 [2]. A decreased level of SPINT2 mRNA and its abnormal splicing were observed, which indicated the loss-of-function of SPINT2 caused by mutation.

After analyzing previous reports on syndromic CTE and SPINT2, we found 35 mutations in SPINT2 from 6 studies (Table 3) [2, 16, 17, 29–31], including 3 noncoding/splicing mutations, 2 frameshift/truncation mutations, and 6 in-frame deletions or missense mutations. Only four of the mutations were reported in two or more patients: c.488A>G (24 patients), c.2T>C (2 patients), c.442C>T (2 patients), and c.502G>A (2 patients). The missense mutation (c.488A>G) was particularly common among seemingly unrelated families with syndromic CTE, which might predict a change in the catalytic domain of the Kunitz-type serine protease inhibitor from an invariantly conserved tyrosine to a cysteine residue (p.Tyr163Cys) [2, 16]. This mutation resulted in reduced inhibitor activity of SPINT2 on the prototype serine protease trypsin. Different mutations might represent different disease subtypes, and to date, all reported syndromic CTE patients with SPINT2 genotypes had at least one of these hypomorphic mutations, and residual SPINT2 activity is required for human development and mouse development.

The presence of SPINT2 is found throughout the gastrointestinal tract of humans and mice [52]. Matriptase, prostasin, SPINT1, and SPINT2 are coexpressed in most developing and adult mammalian epithelia. SPINT2 was thought to target serine proteases, including matriptase and prostasin, which form an interlinked zymogen activation complex that induces the activation of the two proteases [53, 54]. Matriptase (encoded by ST14/MT-SP1/epithin) and prostasin (encoded by CAP-1/PRSS8) are membrane-anchored serine proteases specifically present in the epithelia of small and large intestines [55]. Initially, the oocyte system was used to assess the activity of the intestinal serine proteases matriptase and prostasin and their inhibition induced by SPINT2 and its missense mutant (c.488A>G, p.Tyr163Cys) associated with SCSD. This cellular assay assessed the proteolytic activity of matriptase and prostasin using the epithelial sodium channel ENaC as a reporter gene. The missense mutation of SPINT2 (p.Tyr163Cys) resulted in a loss of inhibitory activity to prostasin and a partial loss of inhibitory activity to matriptase when compared with wild-type SPINT2 [56]. The activity of ENaC is dependent on the matriptase-prostasin system regulated by SPINT2, and ENaC is essential for sodium reabsorption in the large intestine of the distal colon [57]. SPINT2 deficiency in small and large intestinal epithelia could induce a dramatic decrease in the expression of EpCAM and matriptase at late stages of embryonic development, indicating that no environmental triggers (such as microbial colonization or food contact) are required to initiate the molecular event chain that leads to CTE. The deficiency of SPINT2 in the intestine (SPINT2-/- mice) was found to be responsible for a CTE-like phenotype characterized by an inability to gain weight after birth and failure to thrive, accompanied by reduced expression of prostasin and EpCAM protein, followed by a progressive loss of claudin-7, claudin-1, and E-cadherin but an increase in gene and protein expression of claudin-4 [58]. Another study showed that mutations in SPINT2 might result in SCTE due to its reduced inhibitory effect on prostasin or a prostasin-similar protease [52]. In addition, mutations in SPINT2 have been indicated to indirectly induce the loss of EpCAM protein due to proteolysis by activating matriptase [50]. However, the etiology of the syndrome described above in CTE patients identified with SPINT2 mutations has not been clarified.

### 3.3. Unknown Gene Mutation in CTE

As discussed above, the pathogenesis of CTE is associated with mutations in EpCAM or SPINT2. However, in some CTE patients exhibiting isolated diarrhea, neither EpCAM nor SPINT2 mutations were detected. The results of EpCAM and SPINT2 immunostaining in these patients were comparable to the control, supporting the hypothesis of unknown gene mutations in CTE [16], which entails further research.

## 4. Management and Outcome

CTE is a life-threatening disease characterized by persistent severe diarrhea within a few days after birth, leading to rapid dehydration, electrolyte imbalance, and eventually metabolic decompensation. Intractable diarrhea is present independent of breast or formula feeding, and most CTE patients with neonatal diarrhea require total parenteral nutrition (TPN) due to protein energy malnutrition (Table 1), while some infants with a milder phenotype only require partial TPN ranging from 3 to 6 times per week [59, 60]. In any case, careful monitoring should be performed continuously during TPN to ensure appropriate growth. A study on how different mutation types affect prognosis suggested that the frameshift mutation group (particularly c.498insC), the only group with a significant difference, was more likely to require full TPN and was associated with more severe clinical outcomes. In contrast, mutations of splice sites, particularly c.556-14A>G, were more frequently observed in patients requiring partial TPN, suggesting better clinical outcomes [60].

In most patients, CTE causes irreversible intestinal failure (IF). Treatment of patients with IF requires early detection and analysis of irreversible risks. Therefore, in some severe cases, intestinal transplantation (ITx) is required [4, 6, 16, 30, 41, 61, 62], and it is crucial to determine the timing of referral to ITx. The first criteria for ITx was published in the American Society of Transplantation [63]. Since then, changes have been made in the management of IF, bringing about advances in transplantation and improvement in patient survival. Recently, an updated version with new indications for ITx was published. Life-threatening diseases that require indefinite parenteral nutrition, such as CTE, suggest timely ITx for patients with the following situation: 2 admissions to an intensive care unit (after initial recovery from the event inducing IF) because of cardiorespiratory failure (mechanical ventilation or inotrope infusion) due to sepsis or other complications of IF [64].

Generally, management should be based on appropriate parenteral nutrition and timely intestinal transplantation.

## 5. Summary and Future Directions

In the past ten years, we have seen remarkable progress in certain aspects of CTE, such as the pathogenesis and diagnostic methods. Rapidly developing molecular analysis techniques have improved the diagnostic techniques for CTE and reduced invasive and expensive procedures. Existing studies have confirmed that mutations in EpCAM and SPINT2 are the two leading causes of CTE, while a small number of CTE patients did not harbor either EpCAM or SPINT2 mutations, suggesting that other genes might be responsible for these CTE patients, which requires further research. From another perspective, women before or during pregnancy can undergo screening programs for CTE-related pathogenic genes so that early detection, early diagnosis, and early treatment can be achieved.

## Figures and Tables

**Table 1:** Clinical features, gene mutations, and treatments of CTE in reported studies.

Study	Year	CTE number	Symptom	Extraintestinal symptoms	Gene mutation	Number	Treatment	Outcome
Reifen et al. [13]	1994	3	Diarrhea, 2 vomit	NA	NA	NA	PN	1 died
Patey et al. [65]	1997	6	Diarrhea	Unknown	NA	NA	Unknown	Unknown
Goulet et al. [21]	1998	10	Diarrhea	NA	NA	NA	Unknown	Unknown
Beck et al. [61]	2001	1	Diarrhea	NA	NA	NA	PN, ITx	Alive
Cameron and Barnes [59]	2003	1	Diarrhea	NA	NA	NA	PN	Alive
Field [4]	2003	1	Diarrhea, vomit	NA	NA	NA	PN, ITx	Alive
El-Matary et al. [18]	2007	1	Diarrhea, vomit	Skeletal dysplasia	NA	NA	PN	Alive
Bird et al. [28]	2007	3	Diarrhea	Choanal atresia; ophthalmologic, hematologic, and hair abnormalities	NA	NA	Unknown	Unknown
Sivagnanam et al. [7]	2008	5	Diarrhea	NA	EpCAM	4	PN	Unknown
Al-Mayouf et al. [12]	2009	4	Diarrhea	*Chronic arthritis*	EpCAM	1	PN	2 died
Heinz-Erian et al. [2]	2009	16	Diarrhea	Corneal erosions, hypertelorism, choanal atresia, imperforate anus, rectovaginal fistula, short and brittle hair, and mild psychomotor delay	SPINT2	14	PN	11 died
Sivagnanam et al. [14]	2010	1	Diarrhea, emesis	NA	EpCAM	1	PN	Unknown
Ko et al. [15]	2010	2	Diarrhea, vomit	Oligoarticular juvenile rheumatoid arthritis	EpCAM	2	PN	Alive
Roche et al. [6]	2010	15	Diarrhea	Ophthalmic functional disorders, asymptomatic conjunctival hyperemia	NA	NA	PN, ITx	Alive
Salomon et al. [19]	2010	11	Diarrhea	NA	EpCAM	7	PN	Unknown
Sivagnanam et al. [17]	2010	1	Diarrhea	Cholestatic liver disease	SPINT2	1	PN	Alive
Lemale et al. [66]	2011	7	Diarrhea	NA	NA	NA	PN	Alive
Thoeni et al. [44]	2013	1	Diarrhea	NA	EpCAM	1	PN	Alive
Slae et al. [29]	2013	1	Diarrhea	Choanal atresia, hyponatremia, and superficial punctate keratitis	SPINT2	1	PN	Died
Treetipsatit and Hazard [24]	2014	3	Diarrhea	NA	EpCAM	2	Unknown	Unknown
Pêgas et al. [43]	2014	1	Diarrhea	NA	EpCAM	1	PN	Alive
Martin et al. [67]	2014	3	Diarrhea	Unknown	EpCAM	2	Unknown	Unknown
Salomon et al. [16]	2014	57	Diarrhea	Superficial punctuated keratitis, choanal atresia, other atresia, dermatological anomalies, and bone malformations	EpCAM SPINT2	41 12	PN, ITx	8 died
Mueller et al. [42]	2014	1	Diarrhea	NA	EpCAM	1	Unknown	Unknown
Ranganathan et al. [62]	2014	17	Diarrhea	NA	NA	NA	PN, 5 IT	Alive
Haas et al. [22]	2016	2	Diarrhea	NA	EpCAM	2	PN	Alive
d'Apolito et al. [30]	2016	4	Diarrhea	Anal atresia	EpCAM SPINT2	3 1	PN, 3 ITx	2 died
Tang et al. [9]	2016	1	Diarrhea	NA	EpCAM	1	PN	Alive
Azzopardi et al. [32]	2016	1	Diarrhea	Chronic arthritis	EpCAM	1	PN	Alive
AlMahamed and Hammo [41]	2017	4	Diarrhea	NA	EpCAM	4	PN, 2 ITx	2 died
Bodian et al. [26]	2017	2	Diarrhea	NA	EpCAM	1	PN	1 died
Tan et al. [23]	2017	1	Diarrhea	NA	EpCAM	1	PN	Alive
Shakhnovich et al. [25]	2018	1	Diarrhea, emesis	NA	EpCAM	1	PN	Alive
Hirabayashi et al. [31]	2018	1	Diarrhea, vomit	Cleft lip and palate, corneal erosions, optic nerve coloboma, and intermittent exotropia	SPINT2	1	PN	Alive
Pathak et al. [60]	2019	17	Diarrhea	NA	EpCAM	17	PN	Alive
Holt-Danborg et al. [52]	2019	3	Diarrhea	Choanal atresia, enterocutaneous fistula, atrial septal defect, cleft lip and palate, and toe abnormalities	SPINT2	3	PN	Alive
Total		204						

CTE: congenital tufting enteropathy; PN: parenteral nutrition; ITx: intestinal transplantation.

**Table 2:** Quantitative distribution of EPCAM mutations identified in CTE patients.

Mutations		Patients with mutation				Total
Chromosomal deletion	del Ex1-4	1 (Salomon et al. [16])				1
	del Ex1-7	1 (Salomon et al. [16])				1
	del EcPAM	1 (Salomon et al. [16])	1 (AlMahamed and Hammo [41])			2
Noncoding/splicing	c.426-1G>A	1 (Sivagnanam et al. [7])	1 (Shakhnovich et al. [25])			2
	c.491+1G>A	2 (Sivagnanam et al. [7])	1 (Ko et al. [15])	1 (Mueller et al. [42])	5 (Pathak et al. [60])	9
	c.492-5T>C	1 (Salomon et al. [16])	1 (Pathak et al. [60])			2
	c.492-2A>G	4 (Salomon et al. [16])	2 (Salomon et al. [19])			6
	c.492-1G>A	1 (Salomon et al. [16])	1 (Pathak et al. [60])			2
	c.555+1G>C	3 (Salomon et al. [16])	1 (Pathak et al. [60])			4
	c.556-14A>G	1 (Sivagnanam et al. [7])	8 (Salomon et al. [16])	2 (d'Apolito et al. [30])		15
		1 (Bodian et al. [26])	2 (Pathak et al. [60])	1 (Pêgas et al. [43])		
	c∗118T>C	1 (Pathak et al. [60])				1
Frameshift/truncation	c.38_62dup25	1 (Tan et al. [23])	1 (Pathak et al. [60])			2
	c.139C>T	1 (Salomon et al. [16])				1
	c.227C>G	1 (Thoeni et al. [44])	2 (Salomon et al. [16])	1 (Pathak et al. [60])		4
	c.265C>T	1 (Mueller et al. [25])				1
	c.316A>T	1 (Ko et al. [15])				1
	c.321delC	1 (Salomon et al. [16])				1
	c.352_368del	2 (Salomon et al. [16])				2
	c.412C>T	1 (Sivagnanam et al. [14])	1 (AlMahamed and Hammo [41])			2
	c.467delC	1 (Salomon et al. [16])				1
	c.579delT	2 (Pathak et al. [60])				2
	c.540delT	2 (Pathak et al. [60])				2
	c.589C>T	1 (Pathak et al. [60])				1
	c.654delA	2 (Salomon et al. [16])	2 (d'Apolito et al. [30])			4
	c.498insC	1 (Al-Mayouf et al. [12])	6 (Salomon et al. [19])	12 (Salomon et al. [16])	2 (AlMahamed and Hammo [41])	21
Missense mutation/in-frame deletion	c.1A>C	1 (Pathak et al. [60])				1
	c.48_68del21	1 (Pathak et al. [60])				1
	c.113G>A	1 (Pathak et al. [60])				1
	c.267G>C	1 (Pathak et al. [60])				1
	c.307G>A	1 (Salomon et al. [16])	1 (Tang et al. [30])	1 (Pathak et al. [60])		3
	c.314T>G	1 (Salomon et al. [16])				1
	c.359A>T	1 (Salomon et al. [16])				1
	c.380C>T	1 (Salomon et al. [16])				1
	c.437T>A	1 (d'Apolito et al. [30])				1
	c.509_511delTCA	1 (Pathak et al. [60])				1
	c.757G>A	1 (Pathak et al. [60])				1
Total						103

**Table 3:** Quantitative distribution of SPINT2 mutations identified in CTE patients.

Mutations		Patients with mutation				Total
Noncoding/splicing	c.593-1G>A	5 (Heinz-Erian et al. [2])				1
	c.337+2T>C	1 (Heinz-Erian et al. [2])				1
	c.553+2T>A	1 (Heinz-Erian et al. [2])				1
Frameshift/truncation	c.172dupG	1 (Salomon et al. [16])				1
	*c.166_167dupTA*	1 (Hirabayashi et al. [31])				1
Missense mutation	c.488A>G	9 (Heinz-Erian et al. [2])	1 (Sivagnanam et al. [17])	1 (Slae et al. [29])	9 (Salomon et al. [16])	24
		2 (Holt-Danborg et al. [52])	1 (d'Apolito et al. [30])	1 (Hirabayashi et al. [31])		
	c.1A>T	1 (Heinz-Erian et al. [2])				1
	c.2T>C	2 (Salomon et al. 16])				2
	c.247G>T	1 (Salomon et al. [16])				1
	c.442C>T	2 (Salomon et al. [16])				2
	c.502G>A	2 (Salomon et al. [16])				2
	c.477A>G	1 (Holt-Danborg et al. [52])				1
	c.481T>G	1 (Holt-Danborg et al. [52])				1
Total						39

## Data Availability

All data of this study could be obtained by emailing the corresponding author.

## Abbreviations

CDD: Congenital diarrheal disorders
CTE: Congenital tufting enteropathy
TPN: Total parenteral nutrition
EPCAM: Epithelial cell adhesion molecule
SPINT2: Serine Peptidase Inhibitor Kunitz Type 2
SPK: Superficial punctuated keratitis
CSD: Congenital sodium diarrhea
SCTE: Syndromic form of CTE
SNP: Single nucleotide polymorphism
HIA-2: Hepatocyte growth factor activator inhibitor type 2
IF: Intestinal failure
ITx: Intestinal transplantation.
